# Supplementary material for: Effectiveness and safety of psychosocial interventions for the treatment of cannabis use disorder: A systematic review and meta‐analysis
Source: Addiction. 2025 May 2;120(11):2181–201. doi: 10.1111/add.70084 (PMC12529236; doi:10.1111/add.70084)
Supplement: Supplementary file 1 — Data S1. Intervention groupings [file ADD-120-2181-s006.docx]

# Supporting Information 1. Intervention groupings

Here we provide a description of the intervention types and groupings. Abbreviations are those used in the main article, tables and forest plots and throughout the Supporting Information documents. References relating to this Supporting Information are at the end of this document.

*MET-CBT*

Therapies primarily using cognitive-behavioural techniques such as cognitive restructuring, behavioural self-monitoring, coping skills, problem solving and decision-making skills. The majority of studies (n=11) implemented cognitive-behavioural therapy (CBT) targeting issues related to cannabis use (e.g. cognitive restructuring and skills training to understand patterns of use and identify high risk situations, deal with craving or lapses, maintain abstinence, promote assertiveness or manage negative emotions). CBT was delivered in combination with elements of motivation enhancement therapy (MET), such as motivational interviewing and developing commitment to change, usually for the first 1-2 treatment sessions. One study used CBT to target insomnia related to cannabis use, without explicit reference to MET.^1^ However, this grouping was still considered appropriate as the vast majority of intervention time in combined MET-CBT interventions was CBT. Other interventions included in the CBT category were a combination of MET and behavioural therapy focusing on coping skills,^2^ and relapse prevention using MET, cognitive restructuring, and skills training focused on cannabis use.^3,4^ Note that one of the MET-CBT interventions was described as treatment-as-usual that differed between the sites of a multi-centre trial^5^ but the intervention for each site included common MET and CBT elements and individual substance abuse counselling.
Duration: 1.5-6 months (mean [M]=2.95, standard deviation [SD]=1.09). Number of sessions: 6-14 (M=9.86, SD=2.48).

*MET-CBT-affect*

Two studies evaluated MET-CBT-based interventions with a specific emphasis on affect management: integrated cannabis and anxiety reduction treatment (ICART)^6^ and affect management treatment (AMT).^7^ These treatments targeted false safety behaviours, avoidance, negative urgency, distress intolerance and misappraisal.
Duration: 3 months. Number of sessions: 12.

*DBT/ACT*

Third/fourth-wave psychotherapies included dialectical behavioural therapy (DBT),^8^ acceptance and commitment therapy (ACT)^9^ and mindfulness-based psychoeducation,^10^ implemented in one study each. These interventions included elements of cannabis use focused psychoeducation as well as training in mindfulness, emotion regulation, distress tolerance, interpersonal skills, problem solving and acceptance.
Duration: 1-4 months (M=2.67, SD=1.53). Number of sessions: 8-16 (M=12, SD=4).

*CM-abstinence*

Four studies used contingency management (CM) based on abstinence as a standalone intervention.^11-14^ Participants received lottery draws or vouchers for providing cannabinoid-negative urine specimens, usually starting from week 2 of treatment (allowing for a sufficient wash-out period). The value of potential rewards varied from approximately $1 to $100 USD, and typically increased over the duration of the study, according to the length of continuous abstinence. Providing a urine test positive for cannabinoids reset the rewards to the baseline level. Note that Carroll 2006^12^ used a combination of CM based on abstinence and attendance, however, rewards for each were independent and this intervention was classified as CM-abstinence assuming that it would be a stronger therapeutic component.
Duration: 2-3.5 months (M=2.69, SD=0.69). Number of sessions: 8-29 (M=14.5, SD=9.81).

*MET-CBT + CM-abstinence*

In eight studies, CM-abstinence was delivered in addition to MET-CBT, both as described above.
Duration: 2-3.5 months (M=2.84, SD=0.67). Number of sessions: 8-43 (M=20, SD=15.34).

*MET-CBT + CM-attendance*

CM based on attendance was delivered in addition to MET-CBT in four studies. Participants gained rewards for attending intervention sessions, providing urine samples (regardless of the test result), and/or homework completion. Rewards were lottery draws for prizes ($1-100 USD value, amount of draws escalating with consecutive attendance)^13,15^ or fixed-amount vouchers ($5 USD).^11,16^
Duration: 2-3.5 months (M=3, SD=0.71). Number of sessions: 9-41 (M=19.25, SD=15.31).

*ComReinf*

Community reinforcement focused on reducing environmental contingencies that maintain cannabis use and finding new (or enhancing existing) reinforcers for staying abstinent. Interventions involved existing community resources and developing new positive support systems. They included functional analysis and behavioural skills training (such as communication, problem solving, social skills, vocational training).^17,18^
Duration: 1.5-2.5 months (M=2, SD=0.71). Number of sessions: 6-10 (M=8, SD=2.83).

*MDFT*

We considered multidimensional family therapy (MDFT) as a standalone intervention, distinct from the above categories. MDFT focuses on improving multiple life domains, including adolescents’ developmental and intrapersonal issues, individual functioning of their parents and parenting style, the broader family environment, and community systems (such as school, social services, criminal justice), through blended family and individual therapy, drug counselling, and system-oriented interventions.^5^
Duration: 6 months. Number of sessions: 52.

*Inactive/nonspecific comparators*

Inactive comparator represents waitlist control conditions,^3,6,19-21^ where no intervention was provided to participants during the waitlist period, but they were offered an alternative intervention after that period. None of the included studies used ‘no intervention’ control. Nonspecific comparators aim to control for the common features of therapies such as support or educational content but they do not include training in techniques thought of as being therapeutic. ^22,23^ These included social support,^4^ case management,^14,15^ and sham sleep improvement treatment.^1^ Even though we pre-specified counselling and education as example interventions of interest, the included studies that used these conditions described them as routine care, therefore, we classified them as nonspecific comparators.^8,9,12,18^ However, in some settings, a nonspecific comparator may be described as routine care but be considered an active intervention in another setting. Where possible, classification of interventions and comparators was based on author reported content. If insufficient detail was reported, the trialists’ definition was used (e.g., if ‘standard care’ arm received a course of MET-CBT, it was classified as MET-CBT and not as a nonspecific comparator).^5^
Duration and number of sessions (not applicable to waitlist) were typically matched to the active intervention within the same study.

To provide additional context for the interpretation of the review findings, the boxplots (Figure 1) illustrate participants’ age and intervention duration across different intervention categories. Note that these characteristics are presented by intervention, while in randomized controlled trials, age and intervention duration are features of randomized comparisons. Therefore, Figure 1 does not allow us to infer whether these factors act as effect modifiers within specific pairwise comparisons. The boxplots illustrate, however, that interventions such as MDFT and community reinforcement were only evaluated in adolescents/young adults, and that MDFT lasted longer than other interventions. These features may need to be considered when generalising the review findings to other individuals with CUD or implementing such interventions in practice.


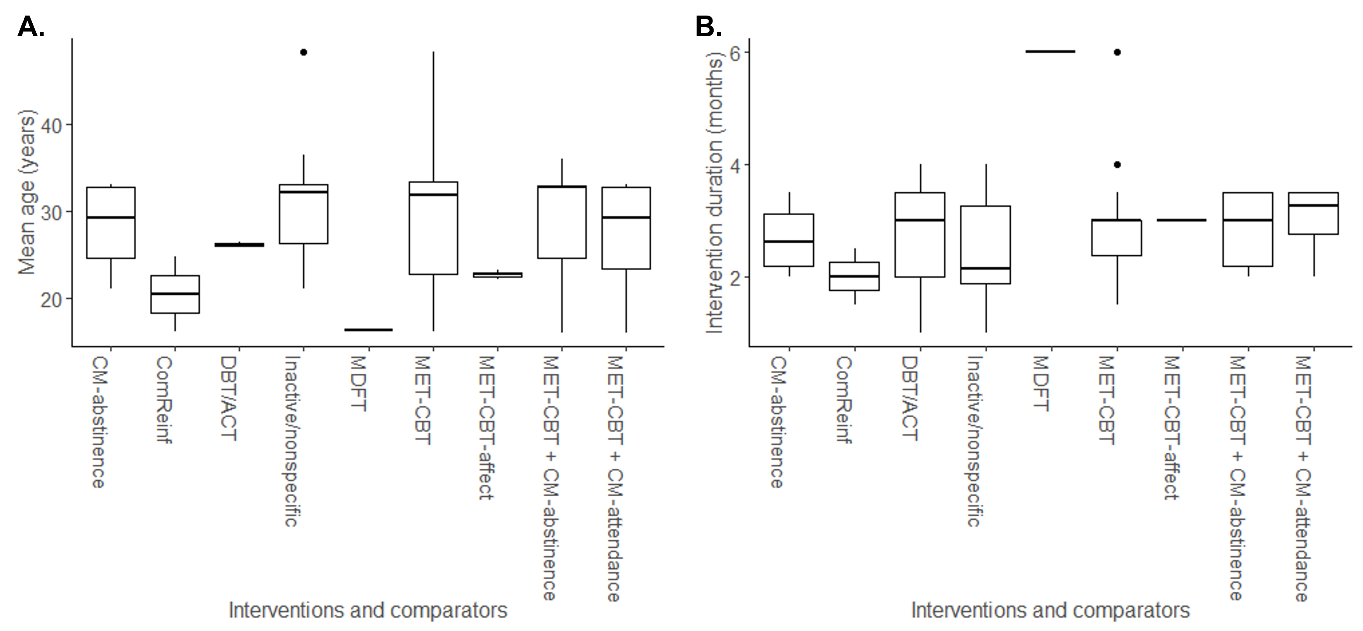


**Figure 1.** Boxplots representing the distributions of (A) age and (B) intervention duration for each intervention category.

## References

1. NCT02102230. CBT-I for Cannabis Use. <https://clinicaltrialsgov/show/NCT02102230>. 2014.

2. Budney AJ, Higgins ST, Radonovich KJ, Novy PL. Adding voucher-based incentives to coping skills and motivational enhancement improves outcomes during treatment for marijuana dependence. Journal of Consulting and Clinical Psychology. 2000;68(6):1051‐61.

3. Stephens RS, Roffman RA, Curtin L. Comparison of extended versus brief treatments for marijuana use. Journal of Consulting and Clinical Psychology. 2000;68(5):898‐908.

4. Stephens RS, Roffman RA, Simpson EE. Treating adult marijuana dependence: a test of the relapse prevention model. Journal of Consulting and Clinical Psychology. 1994;62(1):92‐9.

5. Rigter H, Henderson CE, Pelc I, Tossmann P, Phan O, Hendriks V, et al. Multidimensional family therapy lowers the rate of cannabis dependence in adolescents: a randomised controlled trial in Western European outpatient settings. Drug and Alcohol Dependence. 2013;130(1):85‐93.

6. Buckner JD, Zvolensky MJ, Ecker AH, Schmidt NB, Lewis EM, Paulus DJ, et al. Integrated cognitive behavioral therapy for comorbid cannabis use and anxiety disorders: a pilot randomized controlled trial. Behaviour research and therapy. 2019;115:38‐45.

7. Wolitzky-Taylor K, Glasner S, Tanner A, Ghahremani DG, London ED. Targeting maladaptive reactivity to negative affect in emerging adults with cannabis use disorder: a preliminary test and proof of concept. Behaviour research and therapy. 2022;150:104032.

8. Davoudi M, Allame Z, Foroughi A, Taheri AA. A pilot randomized controlled trial of dialectical behavior therapy (DBT) for reducing craving and achieving cessation in patients with marijuana use disorder: feasibility, acceptability, and appropriateness. Trends in psychiatry and psychotherapy. 2021;43(4):302‐10.

9. Davoudi M, Taheri A, Foroughi A. Effectiveness of Acceptance and Commitment Therapy on Depression, Anxiety and Cessation in Marijuana Use Disorder: a Randomized Clinical Trial. International journal of behavioral sciences. 2021;15(3):194‐200.

10. Budak FK, Akbeniz A, Erkan FM, Gultekin A, Cumurcu HB. The effect of mindfulness-based psychoeducation on negative automatic thoughts and medication adherence in individuals with cannabis use disorder: A randomized controlled trial. International Journal of Mental Health and Addiction. 2024:1-14.

11. Budney AJ, Moore BA, Rocha HL, Higgins ST. Clinical trial of abstinence-based vouchers and cognitive-behavioral therapy for cannabis dependence. Journal of Consulting and Clinical Psychology. 2006;74(2):307‐16.

12. Carroll KM, Easton CJ, Nich C, Hunkele KA, Neavins TM, Sinha R, et al. The use of contingency management and motivational/skills-building therapy to treat young adults with marijuana dependence. Journal of Consulting and Clinical Psychology. 2006;74(5):955‐66.

13. Carroll KM, Nich C, Lapaglia DM, Peters EN, Easton CJ, Petry NM. Combining cognitive behavioral therapy and contingency management to enhance their effects in treating cannabis dependence: less can be more, more or less. Addiction. 2012;107(9):1650‐9.

14. Kadden RM, Litt MD, Kabela-Cormier E, Petry NM. Abstinence rates following behavioral treatments for marijuana dependence. Addictive behaviors. 2007;32(6):1220‐36.

15. Litt MD, Kadden RM, Petry NM. Behavioral treatment for marijuana dependence: randomized trial of contingency management and self-efficacy enhancement. Addictive behaviors. 2013;38(3):1764‐75.

16. Stanger C, Budney AJ, Kamon JL, Thostensen J. A randomized trial of contingency management for adolescent marijuana abuse and dependence. Drug and Alcohol Dependence. 2009;105(3):240‐7.

17. Kaminer Y, Ohannessian CM, Burke RH. Adolescents with cannabis use disorders: adaptive treatment for poor responders. Addictive behaviors. 2017;70:102‐6.

18. Khalily MT, Hussain B, Hallahan B, Irfan S, Ehsan N, Saghir M, et al. Indigenously adapted community reinforcement approach (ia-cra) for cannabis users: A randomized controlled trial. International Journal of Mental Health and Addiction. 2023:1-12.

19. Babor TF. Brief Treatments for Cannabis Dependence: Findings From a Randomized Multisite Trial. Journal of Consulting and Clinical Psychology. 2004;72(3):455-66.

20. Copeland J, Swift W, Roffman R, Stephens R. A randomized controlled trial of brief cognitive-behavioral interventions for cannabis use disorder. Journal of substance abuse treatment. 2001;21(2):55‐64.

21. Hoch E, Bühringer G, Pixa A, Dittmer K, Henker J, Seifert A, et al. CANDIS treatment program for cannabis use disorders: findings from a randomized multi-site translational trial. Drug and Alcohol Dependence. 2014;134:185‐93.

22. Freedland KE, King AC, Ambrosius WT, Mayo-Wilson E, Mohr DC, Czajkowski SM, et al. The selection of comparators for randomized controlled trials of health-related behavioral interventions: recommendations of an NIH expert panel. Journal of Clinical Epidemiology. 2019;110:74-81.

23. Gold SM, Enck P, Hasselmann H, Friede T, Hegerl U, Mohr DC, et al. Control conditions for randomised trials of behavioural interventions in psychiatry: a decision framework. The Lancet Psychiatry. 2017;4(9):725-32.
