# Supplementary material for: Effectiveness and safety of psychosocial interventions for the treatment of cannabis use disorder: A systematic review and meta‐analysis
Source: Addiction. 2025 May 2;120(11):2181–201. doi: 10.1111/add.70084 (PMC12529236; doi:10.1111/add.70084)
Supplement: Supplementary file 3 — Data S3. Search strategies [file ADD-120-2181-s008.docx]

# Supporting Information 3. Search strategies

## Main search strategies

**Ovid MEDLINE(R) ALL** <1946 to June 12, 2024>

1 Marijuana Abuse/ 7227

2 Marijuana Smoking/dt, th 100

3 ((cannabi* or marihuana or marijuana or bhang or ganja or ganjah or hashish) adj3 (problem* use* or overus* or abus* or misus* or dependen* or addict* or use* disorder* or overdos or craving* or cessation* or detox* or withdraw* or abstin*)).tw,id. 6114

4 ((cannabi* or marihuana or marijuana or bhang or ganja or ganjah or hashish) adj3 (high* or heavy or freq*) adj3 (use* or overuse* or risk* or smok*)).tw,kf. 3305

5 (CUD and (cannabi* or marihuana or marijuana or bhang or ganja or ganjah or hashish)).tw,kf. 716

6 ((substance adj2 disorder*) and (cannabi* or marihuana or marijuana or bhang or ganja or ganjah or hashish)).ti,kf,hw. 6925

7 (((relaps* adj1 prevent*) or maintenance) adj5 (cannabi* or marihuana or marijuana or bhang or ganja or ganjah or hashish)).tw,kf. 94

8 or/1-7 17209

9 exp Randomized Controlled Trial/ 616616

10 randomized controlled trial.pt. 615041

11 (randomi#ed or randomi#ation or randomi#ing).tw,kf. 867120

12 (RCT or "at random" or (random* adj3 (administ* or allocat* or assign* or class* or cluster or crossover or cross-over or control* or determine* or divide* or division or distribut* or expose* or fashion or number* or place* or pragmatic or quasi or recruit* or split or substitut* or treat*))).tw,kf. 774763

13 Random Allocation/ 107314

14 randomly.ab. 435329

15 double-blind method/ or single-blind method/ 211424

16 ((single or double or triple or treble) adj2 (blind* or mask* or dummy)).tw,kf. 207158

17 trial.ti. 311159

18 (placebo and control* and trial).ab,kf. 71132

19 (placebo adj5 (control* or group*)).tw,kf. 150098

20 or/9-19 1576036

21 8 and 20 1330

22 ((allocat* or assign*) and (group* or control*)).tw,kf. and (Marijuana Abuse/dt, th or Marijuana Smoking/dt, th) 58

23 21 or 22 1335

24 exp animals/ not humans.sh. 5230386

25 ((animal model* or mouse or mice or murine* or rat or rats or rodent* or muridae or murids or rabbit* or leporine* or leporidae or guineapig* or cavies or caviidae or hamster* or cricetidae or gerbil* or gerbillinae or cat or cats or feline* or felidae or dog or dogs or canine* or canidae or pig or pigs or piglet* or minipig* or swine* or porcine* or suidae or horse or horses or donkey or donkies or burros or asses or equine* or equidae or sheep or lamb or lambs or ovine or ovidae or goat or goats or cow or cows or cattle or bovine* or bovidae or primate* or monkey or monkeys or macaque or macaques or marmoset or marmosets) not human*).ti. 2474021

26 24 or 25 5641233

27 23 not 26 1326

28 (review not randomized controlled trial).pt. 3333282

29 ((systematic review or meta analysis) not randomized controlled trial).pt. 349813

30 27 not (28 or 29) 1175

31 Psychosocial Intervention/ 1169

32 (psychosocial* or psycho-social*).tw,kf. 134314

33 exp Psychotherapy/ 224250

34 (psychotherap* or psycho-therap*).tw,kf. 56450

35 (psychological adj3 (therap* or train* or treat* or trial* or intervention*)).tw,kf. 26785

36 (((behavi* or cognitive) adj3 (therap* or train* or treat* or trial* or intervention*)) or CBT*).tw,kf. 120326

37 behavi* activation.tw,kf. 2671

38 exp Biofeedback, Psychology/ 13356

39 (biofeedback or bio-feedback).tw,kf. 8721

40 exp Counseling/ 49928

41 counsel?ing.tw,kf. 125062

42 exp conditioning, psychological/ or avoidance learning/ 75311

43 (avoidance adj (activit* or learning or train*)).tw,kf. 3273

44 (avoid* adj3 (drug* or cannabi* or marihuana or marijuana or bhang or ganja or ganjah or hashish)).tw,kf. 4456

45 *Motivation/ 34045

46 directive counseling/ or motivational interviewing/ 5106

47 (motivation* adj3 (interview* or enhanc* or therap* or treat* or trial* or intervention*)).tw,kf. 12587

48 (behavio* adj3 (chang* or modif*)).tw,kf. 103651

49 Mindfulness/ 6987

50 (mindful?ness* or (mind adj3 train*)).tw,kf. 14844

51 exp reinforcement, psychology/ or exp reward/ 63218

52 (contingen* adj (manag* or reinforc*)).tw,kf. 1704

53 community reinforcement*.tw,kf. 232

54 exp Psychotherapy, Group/ 28023

55 ((family or families or social) adj3 (support* or therap* or intervention*)).tw,kf. 115303

56 (adaptive adj3 (treatment* or intervention*)).tw,kf. 2175

57 ((monetary or voucher* or prize*) adj5 (incentiv* or contingen* or reinforc* or reward* or treat* or trial* or intervention*)).tw,kf. 4682

58 (vouchers or prizes or rewards).tw,kf. 18571

59 (incentive* or reward* or reinforcement* or motivation* or contingent*).ti,kf. 74896

60 (brief adj3 (therap* or train* or treat* or trial* or intervention*)).tw,kf. 17031

61 Marijuana Abuse/th or Marijuana Smoking/th 566

62 Substance Withdrawal Syndrome/th 1009

63 intervention.ti. or ((cannabi* or marihuana or marijuana or bhang or ganja or ganjah or hashish or addict* or depend* or use* disorder*) adj5 intervention*).ab,kf. 133190

64 or/31-63 1051049

65 30 and 64 582

**Ovid APA PsycInfo** <1806 to June Week 1 2024>

1 *"cannabis use"/ 3527

2 "cannabis use disorder"/ 882

3 (exp Cannabis/ or "Cannabis Use"/) and (Drug Abuse/ or Drug Dependency/ or "Substance Use Disorder"/) 3761

4 ((cannabi* or marihuana or marijuana or bhang or ganja or ganjah or hashish) adj3 (problem* use* or overus* or abus* or misus* or dependen* or addict* or use* disorder* or overdos or craving* or cessation* or detox* or withdraw* or abstin*)).tw,id. 5025

5 ((cannabi* or marihuana or marijuana or bhang or ganja or ganjah or hashish) adj3 (high* or heavy or freq*) adj3 (use* or overuse* or risk* or smok*)).tw,id. 2820

6 (CUD and (cannabi* or marihuana or marijuana or bhang or ganja or ganjah or hashish)).tw,id. 541

7 (((substance adj2 disorder*) or substance use* treatment*) and (cannabi* or marihuana or marijuana or bhang or ganja or ganjah or hashish)).ti,id,hw. 1307

8 (((relaps* adj1 prevent*) or maintenance) adj5 (cannabi* or marihuana or marijuana or bhang or ganja or ganjah or hashish)).tw,id. 94

9 or/1-8 11712

10 randomized controlled trials/ 1077

11 (randomi#ed or randomi#ation or randomi#ing).tw,id. 116232

12 (RCT or "at random" or (random* adj3 (administ* or allocat* or assign* or class* or cluster or crossover or cross-over or control* or determine* or divide* or division or distribut* or expose* or fashion or number* or place* or pragmatic or quasi or recruit* or split or substitut* or treat*))).tw,id. 135759

13 randomly.ab. 86984

14 ((single or double or triple or treble) adj2 (blind* or mask* or dummy)).tw,id. 30071

15 trial.ti. 40759

16 (placebo and control* and trial).ab,id. 8886

17 (placebo adj5 (control* or group*)).tw,id. 23667

18 or/10-17 224211

19 9 and 18 1010

20 ((allocat* or assign*) and (group* or control*)).tw,id. and ((cannabis or marijuana) and (abuse or dependency or "substance use disorder" or "substance use treatment")).hw. 85

21 19 or 20 1036

22 ((animal model* or mouse or mice or murine* or rat or rats or rodent* or muridae or murids or rabbit* or leporine* or leporidae or guineapig* or cavies or caviidae or hamster* or cricetidae or gerbil* or gerbillinae or cat or cats or feline* or felidae or dog or dogs or canine* or canidae or pig or pigs or piglet* or minipig* or swine* or porcine* or suidae or horse or horses or donkey or donkies or burros or asses or equine* or equidae or sheep or lamb or lambs or ovine or ovidae or goat or goats or cow or cows or cattle or bovine* or bovidae or primate* or monkey or monkeys or macaque or macaques or marmoset or marmosets) not human*).ti. 182084

23 21 not 22 1033

24 (("literature review" or "systematic review" or meta analysis) not (clinical trial or empirical study or quantitative study or followup study)).md. 222299

25 23 and 24 69

26 23 not 25 964

27 exp psychosocial interventions/ 2210

28 (psychosocial* or psycho-social*).mp. 140248

29 exp psychotherapy/ 227225

30 (psychotherap* or psycho-therap*).mp. 219754

31 (psychological adj3 (therap* or train* or treat* or trial* or intervention*)).mp. 31119

32 exp behavior therapy/ 114739

33 (((behavi* or cognitive) adj3 (therap* or train* or treat* or trial* or intervention*)) or CBT*).mp. 151003

34 behavi* activation.mp. 3552

35 exp biofeedback/ 6983

36 (biofeedback or bio-feedback).mp. 7732

37 exp counseling/ 85712

38 counsel?ing.mp. 119036

39 exp operant conditioning/ 37838

40 (avoidance adj (activit* or learning or train*)).mp. 14356

41 (avoid* adj3 (drug* or cannabi* or marihuana or marijuana or bhang or ganja or ganjah or hashish)).mp. 787

42 motivation/ 65514

43 motivational interviewing/ 3214

44 (motivation* adj3 (interview* or enhanc* or therap* or treat* or trial* or intervention*)).mp. 13265

45 exp behavior modification/ 27653

46 exp behavior change/ 16564

47 (behavio* adj3 (chang* or modif*)).mp. 83572

48 readiness to change/ 1878

49 mindfulness/ 13493

50 (mindful?ness* or (mind adj3 train*)).mp. 22867

51 exp reinforcement/ 59486

52 exp rewards/ 21330

53 exp contingency management/ 3480

54 (contingen* adj (manag* or reinforc*)).mp. 4145

55 community reinforcement*.tw,id. 380

56 Family Therapy/ or Family Intervention/ 27274

57 social support/ 46216

58 ((family or families or social) adj3 (support* or therap* or intervention*)).mp. 183539

59 (adaptive adj3 (treatment* or intervention*)).mp. 846

60 ((monetary or voucher* or prize*) adj5 (incentiv* or contingen* or reinforc* or reward* or treat* or trial* or intervention*)).mp. 6636

61 (vouchers or prizes or rewards).mp. 36528

62 (incentive* or reward* or reinforcement* or motivation* or contingent*).ti,id. 126718

63 brief interventions/ 458

64 (brief adj3 (therap* or train* or treat* or trial* or intervention*)).mp. 16812

65 exp "substance use treatment"/ or addiction treatment/ 34900

66 exp Intervention/ 148956

67 intervention.ti. or ((cannabi* or marihuana or marijuana or bhang or ganja or ganjah or hashish or addict* or depend* or use* disorder*) adj5 intervention).mp. 63036

68 or/27-67 1169243

69 26 and 68 507

**Central Register of Controlled Trials (CENTRAL) on the Cochrane Library** (Issue 6, 2024; searched 12-June-2024)

ID Search Hits

#1 MeSH descriptor: [Marijuana Abuse] this term only 849

#2 MeSH descriptor: [Marijuana Smoking] this term only and with qualifier(s): [drug therapy - DT, therapy - TH] 51

#3 ((cannabi* or marihuana or marijuana or bhang or ganja or ganjah or hashish) NEAR/3 ((problem* NEXT use*) or overus* or abus* or misus* or dependen* or addict* or (use* NEXT disorder*) or overdos or craving* or cessation* or detox* or withdraw* or abstin*)):ti,ab,kw 1604

#4 ((cannabi* or marihuana or marijuana or bhang or ganja or ganjah or hashish) NEAR/3 (high* or heavy or freq*) NEAR/3 (use* or overuse* or risk* or smok*)):ti,ab,kw 403

#5 (CUD and (cannabi* or marihuana or marijuana or bhang or ganja or ganjah or hashish)):ti,ab,kw 167

#6 ((substance NEAR/2 disorder*) and (cannabi* or marihuana or marijuana or bhang or ganja or ganjah or hashish)):ti,ab,kw 945

#7 (((relaps* NEAR/2 prevent*) or maintenance) NEAR (cannabi* or marihuana or marijuana or bhang or ganja or ganjah or hashish)):ti,ab,kw 46

#8 #1 or #2 or #3 or #4 or #5 or #6 or #7 2346

#9 (psychosocial* or psycho-social*):ti,ab,kw 22061

#10 MeSH descriptor: [Psychotherapy] explode all trees 35556

#11 (psychotherap* or psycho-therap*):ti,ab,kw 17762

#12 (psychological NEAR (therap* or train* or treat* or trial* or intervention*)):ti,ab,kw 21766

#13 (((behavi* or cognitive) NEAR (therap* or train* or treat* or trial* or intervention*)) or CBT*):ti,ab,kw 93878

#14 (behavi* NEXT activation):ti,ab,kw 1405

#15 MeSH descriptor: [Biofeedback, Psychology] explode all trees 2172

#16 (biofeedback or bio-feedback):ti,ab,kw 4551

#17 MeSH descriptor: [Counseling] explode all trees 7565

#18 (counseling or counselling):ti,ab,kw 27992

#19 MeSH descriptor: [Conditioning, Psychological] explode all trees 1325

#20 MeSH descriptor: [Avoidance Learning] this term only 381

#21 avoidance:ti,ab,kw 8299

#22 (avoid* NEAR (drug* or cannabi* or marihuana or marijuana or bhang or ganja or ganjah or hashish)):ti,ab,kw 894

#23 MeSH descriptor: [Motivation] explode all trees 12337

#24 MeSH descriptor: [Motivational Interviewing] this term only 1354

#25 MeSH descriptor: [Directive Counseling] this term only 472

#26 (motivation* NEAR (interview* or enhanc* or therap* or treat* or trial* or intervention*)):ti,ab,kw 11475

#27 (behavio* NEAR (chang* or modif*)):ti,ab,kw 23055

#28 MeSH descriptor: [Mindfulness] explode all trees 2314

#29 (mindfulness* or mindfullness* or (mind NEAR train*)):ti,ab,kw 10050

#30 MeSH descriptor: [Reinforcement, Psychology] explode all trees 3079

#31 MeSH descriptor: [Reward] explode all trees 1490

#32 (contingen* NEXT (manag* or reinforc*)):ti,ab,kw 1088

#33 community reinforcement*:ti,ab,kw 573

#34 ((family or families or social) NEAR (support* or therap* or intervention*)):ti,ab,kw 31868

#35 (adaptive AND (treatment* or intervention*)):ti,ab,kw 8137

#36 ((monetary or voucher* or prize*) AND (incentiv* or contingen* or reinforc* or reward* or treat* or trial* or intervention*)):ti,ab,kw 3419

#37 (vouchers or prizes or rewards):ti,ab,kw 2599

#38 (incentiv* or reward* or reinforce* or motivat* or contingent*):ti,kw 21427

#39 (brief NEAR (therap* or train* or treat* or trial* or intervention*)):ti,ab,kw 12911

#40 MeSH descriptor: [Marijuana Abuse] explode all trees and with qualifier(s): [therapy - TH] 160

#41 MeSH descriptor: [Marijuana Smoking] explode all trees and with qualifier(s): [therapy - TH] 31

#42 MeSH descriptor: [Substance Withdrawal Syndrome] explode all trees and with qualifier(s): [therapy - TH] 120

#43 intervention*:ti 85301

#44 ((cannabi* or marihuana or marijuana or bhang or ganja or ganjah or hashish or addict* or depend* or use* disorder*) NEAR intervention*):ti,ab,kw 42639

#45 #9 OR #10 OR #11 OR #12 OR #13 OR #14 OR #15 OR #16 OR #17 OR #18 OR #19 OR #20 OR #21 OR #22 OR #23 OR #24 OR #25 OR #26 OR #27 OR #28 OR #29 OR #30 OR #31 OR #32 OR #33 OR #34 OR #35 OR #36 OR #37 OR #38 OR #39 OR #40 OR #41 OR #42 OR #43 OR #44 289344

#46 #8 AND #45 1412

[Trials, n=1397]

#47 #8 NOT #46 934

[Trials, n=931]

## Economic search strategies

**Ovid MEDLINE(R) ALL** <1946 to July 30, 2024>

1 Marijuana Abuse/ 7240

2 Marijuana Smoking/dt, th 100

3 ((cannabi* or marihuana or marijuana or bhang or ganja or ganjah or hashish) adj3 (problem* use* or overus* or abus* or misus* or dependen* or addict* or use* disorder* or overdos or craving* or cessation* or detox* or withdraw* or abstin*)).tw,kf. 6284

4 ((cannabi* or marihuana or marijuana or bhang or ganja or ganjah or hashish) adj3 (high* or heavy or freq*) adj3 (use* or overuse* or risk* or smok*)).tw,kf. 3354

5 (CUD and (cannabi* or marihuana or marijuana or bhang or ganja or ganjah or hashish)).tw,kf. 731

6 ((substance adj2 disorder*) and (cannabi* or marihuana or marijuana or bhang or ganja or ganjah or hashish)).ti,kf,hw. 6936

7 (((relaps* adj1 prevent*) or maintenance) adj5 (cannabi* or marihuana or marijuana or bhang or ganja or ganjah or hashish)).tw,kf. 94

8 or/1-7 17367

9 Psychosocial Intervention/ 1194

10 (psychosocial* or psycho-social*).tw,kf. 135407

11 exp Psychotherapy/ 225117

12 (psychotherap* or psycho-therap*).tw,kf. 56765

13 (psychological adj3 (therap* or train* or treat* or trial* or intervention*)).tw,kf. 27112

14 (((behavi* or cognitive) adj3 (therap* or train* or treat* or trial* or intervention*)) or CBT*).tw,kf. 121488

15 behavi* activation.tw,kf. 2690

16 exp Biofeedback, Psychology/ 13410

17 (biofeedback or bio-feedback).tw,kf. 8797

18 exp Counseling/ 50061

19 counsel?ing.tw,kf. 126120

20 exp conditioning, psychological/ or avoidance learning/ 75393

21 (avoidance adj (activit* or learning or train*)).tw,kf. 3279

22 (avoid* adj3 (drug* or cannabi* or marihuana or marijuana or bhang or ganja or ganjah or hashish)).tw,kf. 4490

23 *Motivation/ 34236

24 directive counseling/ or motivational interviewing/ 5132

25 (motivation* adj3 (interview* or enhanc* or therap* or treat* or trial* or intervention*)).tw,kf. 12690

26 (behavio* adj3 (chang* or modif*)).tw,kf. 104473

27 Mindfulness/ 7102

28 (mindful?ness* or (mind adj3 train*)).tw,kf. 15099

29 exp reinforcement, psychology/ or exp reward/ 63424

30 (contingen* adj (manag* or reinforc*)).tw,kf. 1716

31 community reinforcement*.tw,kf. 234

32 exp Psychotherapy, Group/ 28087

33 ((family or families or social) adj3 (support* or therap* or intervention*)).tw,kf. 116550

34 (adaptive adj3 (treatment* or intervention*)).tw,kf. 2204

35 ((monetary or voucher* or prize*) adj5 (incentiv* or contingen* or reinforc* or reward* or treat* or trial* or intervention*)).tw,kf. 4727

36 (vouchers or prizes or rewards).tw,kf. 18741

37 (incentive* or reward* or reinforcement* or motivation* or contingent*).ti,kf. 75596

38 (brief adj3 (therap* or train* or treat* or trial* or intervention*)).tw,kf. 17138

39 Marijuana Abuse/th or Marijuana Smoking/th 568

40 Substance Withdrawal Syndrome/th 1009

41 intervention.ti. or ((cannabi* or marihuana or marijuana or bhang or ganja or ganjah or hashish or addict* or depend* or use* disorder*) adj5 intervention*).ab,kf. 134558

42 ((acceptance adj2 commitment therap*) or dialectical behavio* therap*).mp. 3410

43 (psychoeducat* or psycho-educat* or problem sol*).mp. 53845

44 or/9-43 1097376

45 8 and 44 3035

46 *Economics/ 10816

47 Value of life/ 5828

48 exp "costs and cost analysis"/ 272007

49 exp economics, medical/ 14440

50 exp "fees and charges"/ 31482

51 exp budgets/ 14234

52 budget*.tw,kf. 38439

53 intervention costs.tw,kf. 931

54 (cost? per adj2 (adolescent or adult or man or woman or male or female)).tw,kf. 214

55 economic*.ti. 62835

56 (cost* adj2 (effective* or utilit* or benefit* or minimi* or unit* or estimat* or variable*)).tw,kf. 241478

57 (value adj2 (money or monetary)).tw,kf. 3243

58 or/46-57 550119

59 exp Health Care Costs/ 73393

60 "Cost of Illness"/ 32712

61 Health Expenditures/ 24890

62 (cost? adj2 (illness or disease or sickness or health care or healthcare or treatment or direct or indirect or medical or resource)).tw,kf. 94847

63 (burden? adj2 economic*).tw,kf. 20068

64 (utili?ation adj2 (health or medical or resource)).tw,kf. 38579

65 (out-of-pocket adj2 (payment? or expenditure? or cost? or spending or expense?)).tw,kf. 7665

66 (expenditure? adj3 (health or direct or indirect)).tw,kf. 12207

67 (healthcare cost* or health care cost* or healthcare utili?ation or health care utili?ation or cost of illness).tw,kf. 57334

68 (cost* adj2 (analy* or outcome or outcomes)).tw,kf. 56842

69 or/59-68 277953

70 quality-adjusted life years/ 16622

71 qaly*.tw,kf. 15700

72 quality adjusted life year*.tw,kf. 18466

73 (eq-5d or eq5d or eq-5 or eq5 or euroqual or euro qual or euro qual5d or euroqual5d or euro qol or euroqol or euro qol5d or euroqol5d or euro quol or euroquol or euro quol5d or euroquol5d or eur qol or eurqol or eur qol5d or eur qol5d or eur?qul or eur?qul5d or euro* quality of life or european qol).ti,ab,kf. 19310

74 (euro* adj3 (5 d or 5d or 5 dimension* or 5dimension* or 5 domain* or 5domain*)).tw,kf. 6604

75 ((hql* or hqol* or h qol* or hrqol* or hr qol* or quality of life) adj2 (increase* or decrease* or improv* or declin* or reduc* or high* or low* or effect or effects or worse or score or scores or change? or impact? or impacted or deteriorate*)).tw,kf. 130675

76 *"quality of life"/ and economics.fs. 2946

77 "quality of life"/ and ((quality or qol) adj3 (improv* or chang*)).tw,kf. 46773

78 (health utility* or utility score* or disutilit*).tw,kf. 4071

79 (utilities or (utilit$ adj3 (score? or value* or health* or cost* or analys* or measur* or disease* or mean or gain or gains or index or indices))).tw,kf. 32289

80 (hui or hui1 or hui-1 or hui2 or hui-2 or hui3 or hui-3).tw,kf. 2121

81 health* year* equivalent*.tw,kf. 40

82 (willingness to pay or time tradeoff or time trade off or tto or standard gamble*).tw,kf. 12543

83 (sf36* or sf-36* or sf 36 or sf6 or sf 6 or sf-6 or sf6d or sf 6d or sf-6d or sf8 or sf-8 or sf 8 or sf12 or sf-12 or sf 12 or sf16 or sf-16 or sf 16 or sf20 or sf-20 or sf 20 or sf thirtysix or sf thirty six).tw,kf. 38549

84 (visual analog* scale* or EQ-VAS).tw,kf. 80643

85 or/70-84 309052

86 exp models, economic/ 16429

87 (markov* or monte carlo).tw,kf. 92313

88 econom* model*.tw,kf. 6332

89 ((value adj2 information analysis) or (expected value adj3 perfect information) or (expected value adj3 sampl* information)).tw,kf. 484

90 (microsimulation? or micro-simulation?).tw,kf. 2331

91 discrete event? simulation?.tw,kf. 1056

92 discrete choice experiment*.tw,kf. 3252

93 or/86-92 116560

94 45 and 58 71

95 45 and 69 45

96 45 and 85 34

97 45 and 93 3

98 94 or 95 or 96 or 97 114

**Ovid Embase** <1974 to 2024 July 30>

1 cannabis addiction/ 12284

2 *cannabis smoking/ 1662

3 ((cannabi* or marihuana or marijuana or bhang or ganja or ganjah or hashish) adj3 (problem* use* or overus* or abus* or misus* or dependen* or addict* or use* disorder* or overdos or craving* or cessation* or detox* or withdraw* or abstin*)).tw,kf. 8928

4 ((cannabi* or marihuana or marijuana or bhang or ganja or ganjah or hashish) adj3 (high* or heavy or freq*) adj3 (use* or overuse* or risk* or smok*)).tw,kf. 4478

5 (CUD and (cannabi* or marihuana or marijuana or bhang or ganja or ganjah or hashish)).tw,kf. 996

6 ((substance adj2 disorder*) and (cannabi* or marihuana or marijuana or bhang or ganja or ganjah or hashish)).ti,kf,hw. 2700

7 (((relaps* adj1 prevent*) or maintenance) adj5 (cannabi* or marihuana or marijuana or bhang or ganja or ganjah or hashish)).tw,kf. 118

8 or/1-7 22326

9 psychosocial intervention/ 2818

10 (psychosocial* or psycho-social*).tw,kf. 185802

11 exp psychotherapy/ 311693

12 (psychotherap* or psycho-therap*).tw,kf. 74720

13 (psychological adj3 (therap* or train* or treat* or trial* or intervention*)).tw,kf. 37940

14 (((behavi* or cognitive) adj3 (therap* or train* or treat* or trial* or intervention*)) or CBT*).tw,kf. 166615

15 behavi* activation.tw,kf. 3309

16 exp biofeedback/ 8865

17 (biofeedback or bio-feedback).tw,kf. 13164

18 exp counseling/ 212280

19 counsel?ing.tw,kf. 184901

20 exp "conditioning (psychology)"/ 2738

21 exp avoidance behavior/ 48060

22 (avoidance adj (activit* or learning or train*)).tw,kf. 3254

23 (avoid* adj3 (drug* or cannabi* or marihuana or marijuana or bhang or ganja or ganjah or hashish)).tw,kf. 6854

24 *motivation/ or incentive/ 38121

25 motivational interviewing/ 7538

26 (motivation* adj3 (interview* or enhanc* or therap* or treat* or trial* or intervention*)).tw,kf. 17437

27 (behavio* adj3 (chang* or modif*)).tw,kf. 129981

28 mindfulness/ or mindfulness meditation/ or mindfulness-based stress reduction/ or mindfulness-based cognitive therapy/ 18498

29 "reinforcement (psychology)"/ 5129

30 *reward/ or monetary reward/ 15444

31 (contingen* adj (manag* or reinforc*)).tw,kf. 2119

32 (contingence management or motivational enhancement therapy).dq. 240

33 community reinforcement*.tw,kf. 291

34 group therapy/ or exp family therapy/ 35024

35 ((family or families or social) adj3 (support* or therap* or intervention*)).tw,kf. 148553

36 (adaptive adj3 (treatment* or intervention*)).tw,kf. 3251

37 ((monetary or voucher* or prize*) adj5 (incentiv* or contingen* or reinforc* or reward* or treat* or trial* or intervention*)).tw,kf. 6122

38 voucher program/ 99

39 (vouchers or prizes or rewards).tw,kf. 21985

40 (incentive* or reward* or reinforcement* or motivation* or contingent*).ti,kf. 85342

41 intervention.ti. or ((cannabi* or marihuana or marijuana or bhang or ganja or ganjah or hashish or addict* or depend* or use* disorder*) adj5 intervention*).ab,kf. 179695

42 (mindful?ness* or (mind adj3 train*)).tw,kf. 18820

43 (brief adj3 (therap* or train* or treat* or trial* or intervention*)).tw,kf. 22350

44 ((acceptance adj2 commitment therap*) or dialectical behavio* therap*).mp. 5818

45 (psychoeducat* or psycho-educat* or problem sol*).mp. 74380

46 or/9-45 1417561

47 8 and 46 4241

48 *economics/ 27998

49 health economics/ 36699

50 exp economic evaluation/ 372502

51 "cost"/ 64837

52 exp fee/ 45491

53 budget/ 35031

54 *finance/ 4149

55 budget*.tw,kf. 50829

56 intervention costs.tw,kf. 1149

57 (cost? per adj2 (adolescent or adult or man or woman or male or female)).tw,kf. 271

58 (cost* adj2 (effective* or utilit* or benefit* or minimi* or unit* or estimat* or variable*)).tw,kf. 329813

59 economic*.ti. 77271

60 (value adj2 (money or monetary)).tw,kf. 4375

61 or/48-60 765125

62 *"health care cost"/ or health care financing/ 64373

63 "cost of illness"/ 21924

64 "cost benefit analysis"/ 97705

65 (cost? adj2 (illness or disease or sickness or health care or healthcare or treatment or direct or indirect or medical or resource)).tw,kf. 150863

66 (burden? adj2 economic*).tw,kf. 32092

67 (utili#ation adj2 (health or medical or resource)).tw,kf. 59923

68 (out-of-pocket adj2 (payment? or expenditure? or cost? or spending or expense?)).tw,kf. 10930

69 (expenditure? adj3 (health or direct or indirect)).tw,kf. 16027

70 (healthcare cost* or health care cost* or healthcare utili?ation or health care utili?ation or cost of illness).tw,kf. 90958

71 (cost* adj2 (analy* or outcome or outcomes)).tw,kf. 88109

72 or/62-71 420490

73 quality adjusted life year/ 38132

74 (quality adjusted life year? or qaly* or qald* or qale* or qtime*).tw,kf. 35637

75 (eq-5d or eq5d or eq-5 or eq5 or euroqual or euro qual or euro qual5d or euroqual5d or euro qol or euroqol or euro qol5d or euroqol5d or euro quol or euroquol or euro quol5d or euroquol5d or eur qol or eurqol or eur qol5d or eur qol5d or eur?qul or eur?qul5d or euro* quality of life or european qol).tw,kf. 34287

76 (euro* adj3 (5 d or 5d or 5 dimension* or 5dimension* or 5 domain* or 5domain*)).tw,kf. 9836

77 ((hql* or hqol* or h qol* or hrqol* or hr qol*) adj2 (increase* or decrease* or improv* or declin* or reduc* or high* or low* or effect or effects or worse or score or scores or change? or impact? or impacted or deteriorate*)).tw,kf. 20421

78 "quality of life"/ and economics/ 3589

79 (multiattribute* or multi attribute*).tw,kf. 1698

80 health utilit*.tw,kf. 4970

81 (utilit* adj2 (value* or cost* or health or analys* or index or indices)).tw,kf. 25861

82 disutilit$.tw,kf. 1385

83 (hsuv or hsuvs).tw,kf. 226

84 (health? year? equivalent? or hye?).tw,kf. 274

85 (hui or hui1 or hui2 or hui3 or hui-1 or hui-2 or hui-3).tw,kf. 3408

86 (hye or hyes).tw,kf. 200

87 rosser.tw,kf. 147

88 exp short form 36/ and (QoL or "quality of life").mp. 39025

89 (sf36$ or sf-36$ or sf 36 or sf6 or sf 6 or sf-6 or sf6d or sf 6d or sf-6d or sf8 or sf-8 or sf 8 or sf12 or sf-12 or sf 12 or sf16 or sf-16 or sf 16 or sf20 or sf-20 or sf 20 or sf thirtysix or sf thirty six).tw,kf. and (QoL or "quality of life").mp. 45288

90 (15d or 15-d or 15 dimension).tw,kf. 8032

91 standard gamble/ or time trade-off method/ or willingness to pay/ 5216

92 standard gamble*.tw,kf. 1242

93 ("time trade off?" or time tradeoff? or tto or timetradeoff?).tw,kf. 3770

94 "willingness to pay".tw,kf. 14467

95 EQ-VAS.tw,kf. 2800

96 or/73-95 173814

97 economic model/ or econometric model/ 4306

98 monte carlo method/ or markov chain monte carlo method/ 56196

99 econom* model*.tw,kf. 8942

100 (markov* or monte carlo).tw,kf. 104593

101 ((value adj2 information analysis) or (expected value adj3 perfect information) or (expected value adj3 sampl* information)).tw,kf. 689

102 (microsimulation? or micro-simulation?).tw,kf. 3548

103 discrete event? simulation?.tw,kf. 1611

104 discrete choice experiment?.tw,kf. 4724

105 or/97-104 135178

106 47 and 61 107

107 47 and 72 72

108 47 and 96 23

109 47 and 105 4

110 or/106-109 152
