# Supplementary material for: Effectiveness and safety of psychosocial interventions for the treatment of cannabis use disorder: A systematic review and meta‐analysis
Source: Addiction. 2025 May 2;120(11):2181–201. doi: 10.1111/add.70084 (PMC12529236; doi:10.1111/add.70084)
Supplement: Supplementary file 5 — Data S5. Additional comparisons [file ADD-120-2181-s005.docx]

# Supporting Information 5. Additional comparisons

The following pairwise comparisons were prioritized for meta-analyses and reported in the main article: (1) motivation enhancement and cognitive-behavioural therapy (MET-CBT) versus inactive/nonspecific comparators; (2) dialectical behaviour/acceptance and commitment therapies (DBT/ACT) versus inactive/nonspecific comparators; (3) MET-CBT plus affect management (MET-CBT-affect) versus standard MET-CBT; (4) MET-CBT plus abstinence-based contingency management (CM-abstinence) versus MET-CBT alone; (5) MET-CBT plus CM-abstinence versus MET-CBT plus attendance-based CM (CM-attendance); (6) multidimensional family therapy (MDFT) versus MET-CBT; and (7) community reinforcement versus other active/nonspecific comparators. Study-level effect estimates for the remaining comparisons are reported in Table 1. Numbering of tables is specific to this Supporting Information document. References relating to this Supporting Information are at the end of this document.

**Table 1.** Study-level effect estimates for additional comparisons not included in the meta-analyses, for outcomes assessed at the end of treatment

| **Comparison** | **Study** | **Study arms** | **Relative effect estimate [95% CI]** |
| --- | --- | --- | --- |
| CM-abstinence vs Inactive/nonspecific | Kadden 2007^1^ | CM-ab vs NS | Continuous abstinence: OR 2.12 [0.76; 5.91]  Completion of treatment: OR 1.85 [0.53; 6.53]  Frequency of cannabis use: RoM 0.55 [0.40; 0.76]  Duration of continuous abstinence: RoM 1.63 [1.02; 2.62] |
|  | Carroll 2006^2^ | CM-ab/at vs NS | Completion of treatment: OR 0.67 [0.10; 4.27]  Frequency of cannabis use: RoM 1.00 [0.71; 1.41]  Duration of continuous abstinence: RoM 2.25 [0.86; 5.89] |
| MET-CBT + CM-abstinence vs CM-abstinence | Budney 2006^3^ | MET-CBT+CM-ab vs CM-ab | Continuous abstinence: OR 1.00 [0.33; 3.03]  Point abstinence: OR 1.00 [0.33; 3.03]  Completion of treatment: OR 1.63 [0.41; 6.47]  Frequency of cannabis use: RoM 0.86 [0.54; 1.35] |
|  | Carroll 2006^2^ | MET-CBT+CM-ab-at vs CM-ab-at | Completion of treatment: OR 1.50 [0.23; 9.61]  Frequency of cannabis use: RoM 0.71 [0.48; 1.06]  Duration of continuous abstinence: RoM 1.22 [0.71; 2.10] |
|  | Carroll 2012^4^ | MET-CBT+CM-ab vs CM-ab | Completion of treatment: OR 0.43 [0.08; 2.43]  Frequency of cannabis use: RoM 1.55 [0.92; 2.60]  Duration of continuous abstinence: RoM 0.85 [0.52; 1.41] |
|  | Kadden 2007^1^ | MET-CBT+CM-ab vs CM-ab | Continuous abstinence: OR 0.81 [0.33; 2.00]  Completion of treatment: OR 1.18 [0.28; 4.96]  Frequency of cannabis use: RoM 1.19 [0.85; 1.67]  Duration of continuous abstinence: RoM 0.91 [0.60; 1.39] |
| MET-CBT vs CM-abstinence | Carroll 2006^2^ | MET-CBT vs CM-ab-at | Completion of treatment: OR 0.60 [0.13; 2.73]  Frequency of cannabis use: RoM 1.00 [0.71; 1.41]  Duration of continuous abstinence: RoM 0.72 [0.36; 1.43] |
|  | Carroll 2012^4^ | MET-CBT vs CM-ab | Completion of treatment: OR 0.14 [0.03; 0.70]  Frequency of cannabis use: RoM 1.12 [0.64; 1.95]  Duration of continuous abstinence: RoM 1.06 [0.69; 1.64] |
|  | Kadden 2007^1^ | MET-CBT vs CM-ab | Continuous abstinence: OR 0.54 [0.20; 1.45]  Completion of treatment: OR 0.73 [0.20; 2.75]  Frequency of cannabis use: RoM 1.48 [1.06; 2.06]  Duration of continuous abstinence: RoM 0.68 [0.43; 1.08] |
| MET-CBT + CM-attendance vs MET-CBT | Carroll 2012^4^ | MET-CBT+CM-at vs MET-CBT | Completion of treatment: OR 2.45 [0.80; 7.49]  Frequency of cannabis use: RoM 0.97 [0.61; 1.53]  Duration of continuous abstinence: RoM 0.95 [0.66; 1.36] |
| CBT, cognitive-behavioural therapy; CI, confidence interval; CM, contingency management; CM-ab, contingency management based on abstinence; CM-at, contingency management based on attendance; CM-ab-at, contingency management based on abstinence and attendance; MET, motivation enhancement therapy; NS, nonspecific treatment; OR, odds ratio; RoM, ratio of means. | | | |

## References

1. Kadden RM, Litt MD, Kabela-Cormier E, Petry NM. Abstinence rates following behavioral treatments for marijuana dependence. Addictive behaviors. 2007;32(6):1220‐36.

2. Carroll KM, Easton CJ, Nich C, Hunkele KA, Neavins TM, Sinha R, et al. The use of contingency management and motivational/skills-building therapy to treat young adults with marijuana dependence. Journal of Consulting and Clinical Psychology. 2006;74(5):955‐66.

3. Budney AJ, Moore BA, Rocha HL, Higgins ST. Clinical trial of abstinence-based vouchers and cognitive-behavioral therapy for cannabis dependence. Journal of Consulting and Clinical Psychology. 2006;74(2):307‐16.

4. Carroll KM, Nich C, Lapaglia DM, Peters EN, Easton CJ, Petry NM. Combining cognitive behavioral therapy and contingency management to enhance their effects in treating cannabis dependence: less can be more, more or less. Addiction. 2012;107(9):1650‐9.
