# Supplementary material for: Effectiveness and safety of psychosocial interventions for the treatment of cannabis use disorder: A systematic review and meta‐analysis
Source: Addiction. 2025 May 2;120(11):2181–201. doi: 10.1111/add.70084 (PMC12529236; doi:10.1111/add.70084)
Supplement: Supplementary file 6 — Data S6. Certainty of evidence (GRADE) criteria [file ADD-120-2181-s011.docx]

# Supporting Information 6. Certainty of evidence (GRADE) criteria

To assess the certainty of evidence, we followed the GRADE approach.^1^ We outline the domains we considered and the steps taken to assess certainty within each domain. References relating to this Supporting Information are included at the end of this document.

#### Study limitations (risk of bias)

We assessed risk of bias using the Risk of Bias 2 (RoB 2)^2^ tool for each study that contributed data for a specific outcome. If we had concerns regarding the risk of bias in the studies included in an analysis, the certainty of the evidence was reduced by either one or two levels, depending on the strength of concern, the number (and weight) of the studies affected.

#### Inconsistency (heterogeneity)

We assessed inconsistency using visual inspection of the forest plots (assessing the overlap in confidence intervals, and the direction and magnitude of effects in the individual studies) and the I^2^ value. If a single study was included, we did not reduce the certainty of the evidence for inconsistency.

#### Indirectness

We considered the population, interventions, comparators and outcomes (PICO) included in every trial contributing to a specific outcome. If the PICO for individual studies differed slightly from that assessed in the review we considered the weight of these studies in the analysis. If studies with concerns over indirectness had a substantial impact on the analysis results we reduced the certainty of the evidence. Examples included recruitment of some adolescents aged below 16 years, or inclusion of specific populations of people with cannabis use disorder (CUD) (for example, only those with insomnia or anxiety disorders).

#### Imprecision

There are no established thresholds representing minimally important differences for the outcomes considered in this review. Therefore, we applied thresholds for what could constitute a clinically meaningful change, that had been derived in consultation with topic experts.

For dichotomous outcomes (point abstinence, continuous abstinence, completion of treatment) we assumed an event rate in the control group, and specified an event rate in the intervention group that we considered would represent an important difference between groups. For abstinence outcomes, we assumed a control group rate of 20%, and an intervention group rate of 30%. Sample size calculations indicated that each group would require approximately 293 participants, to detect this difference (with power of 0.8 at the 0.05 level). We therefore used a total sample size of 586 participants for the optimal information size (OIS). For completion of treatment, we assumed a control group rate of 70%, and an intervention group rate of 80%. This required a sample size of 293 participants for each group, therefore we used 586 as the OIS.

If the OIS was not reached then we reduced the certainty of the evidence by one level. If the sample size was extremely small (<100 participants) then we reduced the certainty of the evidence by two levels.

If the OIS was reached then we considered the breadth of the confidence intervals, taking both relative and absolute effects into account. We assumed a change in odds ratio of 25% would represent an important difference, i.e. if the confidence intervals for the odds ratio (OR) crossed the thresholds of either 0.8 or 1.25 we considered reducing the certainty of the evidence due to imprecision by one level. If both thresholds were crossed we considered reducing the certainty of the evidence by two levels. However, we also considered the change in absolute risk before applying these rules. Where large changes in relative risk (as assessed by confidence intervals surrounding the OR) did not equate to substantial changes in absolute risk, we did not reduce the certainty of the evidence.

For continuous data, we also estimated an OIS using assumed control group rates. For duration of continuous abstinence, we assumed a control group rate of 7 days, an intervention group rate of 14 days, and a standard deviation (SD) of 14 days (note the large SD in the data reported). This gave an estimated total sample size of 126 participants, which was used for the OIS. Similarly, for frequency of use we assumed a control group rate of 0.5 (proportion of days using), an intervention rate of 0.25, and a SD of 0.3, which gave an estimated total sample size of 46 participants. For quantity of cannabis use we assumed use of 2 joints per day in the control group, and 1 joint per day in the intervention group, with a SD of 2, which gave an estimated total sample size of 126 participants, which was used for the OIS.

Again, if the OIS was not reached then the certainty of the evidence was reduced by one level. If the OIS was reached then we considered the breadth of the confidence intervals. For continuous outcomes, we assumed that halving of either the quantity or frequency of use would represent an important difference, i.e. if the confidence intervals for the ratio of means (RoM) crossed the thresholds of either 0.5 or 2 we considered reducing the certainty of the evidence due to imprecision by one level. If both thresholds were crossed we considered reducing the certainty of the evidence by two levels.

Only one study reported on cannabis craving, using the Marijuana Craving Questionnaire short-form.^3^ This questionnaire has 12 domains, each rated with a Likert scale on a score of 1-7, giving a total range of scores from 12-84, with higher scores representing worse cravings. We are not aware of an agreed minimally important difference for this questionnaire, therefore assumed that a change of 4 points would be clinically meaningful (the reported standard deviation was approximately 8 points, so this magnitude of change represents approximately half of the sample standard deviation). The required sample size for power of 0.8 at the 0.05 significance level was estimated at 126 participants.

#### Publication bias

We also considered the risk of publication bias in the results presented. Meta-analyses did not include a sufficient number of studies to test funnel plot asymmetry, making it challenging to infer about missing unpublished evidence. Our searches were comprehensive, which reduces the likelihood that studies were omitted from the review. However, we did note that many studies did not report data for all outcomes of interest in this review. This may simply be because these outcomes were not assessed, but also raises the possibility of selective reporting, where authors have deliberately withheld data from publication. Most of the studies included did not report full trial registrations or protocols, and we were therefore unable to assess the trialists intentions. In addition, the trial registrations that were available did not typically report in sufficient detail to be able to establish whether all outcomes were fully reported. To further investigate the possibility of missing evidence, we considered the risk of bias due to missing evidence in the synthesis (ROB-ME).^4^ Based on this framework, we identified three missing evidence scenarios that could apply to the meta-analyses in this review:

1. Meta-analyses of completion of treatment. The number of participants in each arm who completed the end of treatment assessment should be known and reported in all trials reporting any outcomes at this timepoint. Evidence was considered to be missing from four studies;^5-8^
2. Meta-analyses for point abstinence at the end of treatment. Results for point abstinence at the end of treatment should be available if a study measured continuous abstinence at the end of treatment. Evidence was considered to be missing for six studies;^7-12^
3. Meta-analyses for the quantity of cannabis used. Where summary statistics for quantity of cannabis use at the end of treatment were reported only globally (across all groups), these statistics should also be available by intervention group. Evidence was considered to be missing for two studies.^6,10^

There were no indications that other synthesised outcomes were measured but not reported in the included studies.

Serious concerns regarding publication bias were noted if there was high risk of bias due to missing evidence, that is, if a large proportion of evidence was missing from a meta-analysis. This was estimated as the number of participants in studies with missing evidence (that would otherwise be eligible for a particular meta-analysis) divided by the total number of participants in studies included in a meta-analysis plus studies with missing evidence.

## References

1. Guyatt GH, Oxman AD, Vist GE, Kunz R, Falck-Ytter Y, Alonso-Coello P, et al. GRADE: an emerging consensus on rating quality of evidence and strength of recommendations. British Medical Journal. 2008;336(7650):924-6.

2. Sterne JAC, Savović J, Page MJ, Elbers RG, Blencowe NS, Boutron I, et al. RoB 2: a revised tool for assessing risk of bias in randomised trials. British Medical Journal. 2019;366.

3. Davoudi M, Allame Z, Foroughi A, Taheri AA. A pilot randomized controlled trial of dialectical behavior therapy (DBT) for reducing craving and achieving cessation in patients with marijuana use disorder: feasibility, acceptability, and appropriateness. Trends in psychiatry and psychotherapy. 2021;43(4):302‐10.

4. Page MJ, Sterne JAC, Boutron I, Hróbjartsson A, Kirkham JJ, Li T, et al. ROB-ME: a tool for assessing risk of bias due to missing evidence in systematic reviews with meta-analysis. British Medical Journal. 2023;383:e076754.

5. Budney AJ, Higgins ST, Radonovich KJ, Novy PL. Adding voucher-based incentives to coping skills and motivational enhancement improves outcomes during treatment for marijuana dependence. Journal of Consulting and Clinical Psychology. 2000;68(6):1051‐61.

6. Hoch E, Bühringer G, Pixa A, Dittmer K, Henker J, Seifert A, et al. CANDIS treatment program for cannabis use disorders: findings from a randomized multi-site translational trial. Drug and Alcohol Dependence. 2014;134:185‐93.

7. Litt MD, Kadden RM, Petry NM. Behavioral treatment for marijuana dependence: randomized trial of contingency management and self-efficacy enhancement. Addictive behaviors. 2013;38(3):1764‐75.

8. Stephens RS, Roffman RA, Simpson EE. Treating adult marijuana dependence: a test of the relapse prevention model. Journal of Consulting and Clinical Psychology. 1994;62(1):92‐9.

9. Babor TF. Brief Treatments for Cannabis Dependence: Findings From a Randomized Multisite Trial. Journal of Consulting and Clinical Psychology. 2004;72(3):455-66.

10. Kadden RM, Litt MD, Kabela-Cormier E, Petry NM. Abstinence rates following behavioral treatments for marijuana dependence. Addictive behaviors. 2007;32(6):1220‐36.

11. Stephens RS, Roffman RA, Curtin L. Comparison of extended versus brief treatments for marijuana use. Journal of Consulting and Clinical Psychology. 2000;68(5):898‐908.

12. Litt MD, Kadden RM, Tennen H, Petry NM. Individualized assessment and treatment program (IATP) for cannabis use disorder: randomized controlled trial with and without contingency management. Psychology of addictive behaviors. 2020;34(1):40‐51.
